# Development and Validation of a Prognostic Nomogram in Lung Cancer With Obstructive Sleep Apnea Syndrome

**DOI:** 10.3389/fmed.2022.810907

**Published:** 2022-03-18

**Authors:** Wei Liu, Ling Zhou, Dong Zhao, Xiaofeng Wu, Fang Yue, Haizhen Yang, Meng Jin, Mengqing Xiong, Ke Hu

**Affiliations:** ^1^Department of Respiratory and Critical Care Medicine, Renmin Hospital of Wuhan University, Wuhan, China; ^2^Department of Respiratory and Critical Care Medicine, Tongji Hospital, Tongji Medical College Huazhong University of Science and Technology, Wuhan, China

**Keywords:** obstructive sleep apnea syndrome, lung cancer, prognosis, nomogram, development

## Abstract

To analyze the prognostic factors and survival rate of lung cancer patients with obstructive sleep apnea (OSA) by nomogram. The nomogram was established by a development cohort (*n* = 90), and the validation cohort included 38 patients. Factors in the nomogram were identified by Cox hazard analysis. We tested the accuracy of the nomograms by discrimination and calibration, and plotted decision curves to assess the benefits of nomogram-assisted decisions. There were significant difference in sex, apnea hypopnea index (AHI), Tumor Node Metastasis (TNM), coronary heart disease, lowest arterial oxygen saturation [LSpO2 (%)], oxygen below 90% of the time [T90% (min)], the percentage of the total recorded time spend below 90% oxygen saturation (TS90%) and oxygen desaturation index (ODI4) between lung cancer subgroup and lung cancer with OSA subgroup (*P* < 0.05). Lung cancer patients with OSA age, AHI, TNM, cancer types, BMI and ODI4 were independent prognostic factor. Based on these six factors, a nomogram model was established. The c-index of internal verification was 0.802 (95% CI 0.767–0.885). The ROC curve analysis for the nomogram show 1-year survival (AUC = 0.827), 3-year survival (AUC = 0.867), 5-year survival (AUC = 0.801) in the development cohort were good accuracy. The calibration curve shows that this prediction model is in good agreement. Decision curve analysis (DCA) suggests that the net benefit of decision-making with this nomogram is higher, especially in the probability interval of <20% threshold. The nomogram can predict the prognosis of patients and guide individualized treatment.

## Introduction

Lung cancer is the leading cause of cancer death in the world, at present, the pathogenesis of lung cancer is not clearly. Sleep apnea syndrome (SAS) is a disease with high incidence rate and serious harm to patient's health, obstructive sleep apnea (OSA) accounts for more than 90% of SAS (1). OSA is an independent risk factor of multiple system chronic diseases such as hypertension, coronary heart disease, arrhythmia and stroke (2), which also effects the development of chronic obstructive pulmonary disease (3). OSA also plays an important role in the occurrence and development of tumors (4), lung cancer is the malignant tumor which most closely related with OSA (5).

Through there are comprehensive treatments of lung cancer, the survival rate has been significantly improved, improving the quality of life, especially sleep quality were important for lung cancer patients. It's not only helps to accelerate the physical and mental recovery, but also increases the body's immunity and resistance. Lung cancer is the most common cancer in OSA patients, OSA is usually develop moderate or severe when diagnosis, lung cancer and intermittent hypoxia, apnea, daytime sleepiness forming a vicious cycle, decreased the quality of life and survival time significantly (6).

Currently, nomogram have been developed in the majority of cancer types (7–9), in this study, we aimed to develop and validate a prognostic nomogram which uses widely available general data and laboratory indicators to improve our ability to predict survival time of lung cancer patients with OSA.

## Methods

### Patient Selection

The retrospectively study included 410 lung cancer patients from 2013 to 2016 in Renmin Hospital of Wuhan University and Tongji Hospital, all subjects received philips YZB/USA 1575-2013 portable sleep recorder to monitor patients' sleep for at least 7 h per night, there are 128 cases diagnosis of OSA, the inclusion criteria were as follows: (1) the pathological diagnosis was confirmed as lung malignant tumor; (2) normal range of blood pressure in patients with hypertension after use of antihypertensive treatment and no hypertension-related complications; after symptomatic treatment, the patients with CHD were stable without complications; the blood glucose level in patients with type 2 diabetes without complications; (3) no drugs possibly influencing the sleep patterns was currently being taken.

Patients suffering from the following diseases or lesions were excluded: respiratory infectious disease, intracranial lesions, pulmonary embolism, rheumatic diseases and other chronic disease diseases that may cause abnormal blood oxygen saturation (10, 11). This study was conducted in accordance with the Declaration of *Helsinki*.

### Laboratory Measurements

All subjects received philips YZB/USA 1575-2013 portable sleep recorder to monitor patients' sleep for at least 7 h per night, all data previously listed was send back, analyzed by computer and corrected artificially. The data about apnea hypopnea index (AHI), oxygen desaturation index (ODI4), lowest arterial oxygen saturation [LSpO2 (%)], oxygen below 90% of the time [T90% (min)], and the percentage of the total recorded time spend below 90% oxygen saturation (TS90%) were obtained. All the data was recorded when the first time of hospitalizations, clinical information was extracted from Electronic Medical Record system. All the patients received routine tests at the first time visit in hospital.

### Follow-Up

Patients were advised to receive regular follow-ups after completion of the primary therapy according to clinical guidelines. Patients were generally follow-up every 3 months in the first 2 years and annually thereafter for patients without evidence of recurrence in the following 3–5 years. OS was defined as the time from the diagnosis of lung cancer to the time of the last follow-up or death, the follow-up deadline was November 1, 2020.

### Statistical Analysis

Statistical analyses were performed by SPSS 25.0 (IBM, Chicago, IL, USA) and R for Windows (version3.4.2, http://www.r-project.org/). The optimal cut-off points in our study were evaluated by minimum *P*-value from log-rank ×2 statistics using the X-tile program (12) and continuous variables were transformed to categorical variables, regression analysis was used to analyze the risk factors in the development cohort, nomogram was formulated based on the results of univariate and multivariate analysis by the package of rms. Study tested the accuracy of the nomograms by discrimination and calibration both in primary and externa validation cohort. The discrimination of the nomogram was measured by Harrell's C-index (C-index). The calibration curve of the nomogram model for the overall survival were formulated. The total points of each patient were calculated according to the established Cox regression model. Survival curves were depicted by the Kaplan–Meier method. A two-sided *P* < 0.05 was considered statistically significant.

## Results

### Clinical Characteristics of All Patients

The clinical characteristics of all patients were evaluated. The characteristics of the 282 lung cancer patients and 128 lung cancer patients with OSA are showed in Table 1. The median age was 59.98 years and only 172 patients (42.0%) were female. Among the 410 patients, there were 73 patients with the small cell carcinoma (17.8%) and 337 patients with the non-small cell carcinoma (82.2%). There was significant difference in sex, AHI, TNM stage, coronary heart disease, LSpO2, T90%, TS90%, and ODI4 between lung cancer subgroup and lung cancer with OSA subgroup (*P* < 0.05).

**Table 1:** Baseline clinical features of all patients [Mean ± SD/No (%)].

**Characteristics**	**Total** **(*n* = 410)**	**Lung cancer** **(*n* = 282)**	**Lung cancer with OSA** **(*n* = 128)**	**Statistics**	***P***
Age, year	59.98 ± 3.22	59.53 ± 3.53	60.98 ± 2.45	1.432^*^	0.853
Sex				6.382^†^	0.011
Male	238 (58.0)	152 (53.9)	86 (67.2)		
Female	172 (42.0)	130 (46.1)	42 (32.8)		
AHI	4.35 ± 2.01	2.54 ± 2.03	9.87 ± 1.98	0.637^*^	0.001
TNM stage				11.860^†^	0.008
I	129 (31.5)	95 (33.7)	34 (26.6)		
II	147 (35.9)	102 (36.2)	45 (35.2)		
III	83 (20.2)	45 (16.0)	38 (29.7)		
IV	51 (12.4)	40 (14.2)	11 (8.5)		
Cancer types				1.115^†^	0.291
Small cell carcinoma	73 (17.8)	54 (19.1)	19 (14.8)		
Non-small cell carcinoma	337 (82.2)	228 (80.9)	109 (85.2)		
BMI	20.88 ± 1.78	20.43 ± 1.54	21.90 ± 2.98	1.445^*^	0.384
Hypertension	128 (31.2)	84 (29.8)	44 (34.4)	0.863^†^	0.353
Diabetes	144 (35.1)	102 (36.2)	42 (32.8)	0.435^†^	0.509
Coronary heart disease	64 (15.6)	37 (13.1)	27 (21.2)	4.249^†^	0.039
Heart rate, beats/min	91.76 ± 20.34	91.43 ± 21.54	92.34 ± 17.92	1.432^*^	0.758
KPS	80.23 ± 12.44	82.34 ± 13.54	76.34 ± 8.342	0.552^*^	0.817
Smoking history	167 (40.7)	110 (39.0)	57 (44.5)	1.113^†^	0.292
LSpO2 (%)	78.32 ± 10.23	87.32 ± 15.64	69.43 ± 6.31	1.954^*^	0.009
T90%, min	56.34 ± 3.41	23.43 ± 1.65	91.23 ± 4.43	1.943^*^	0.001
TS90%, %	9.32 ± 2.01	1.32 ± 1.03	22.41 ± 2.89	3.215^*^	0.001
ODI4	0.45 ± 0.19	0.23 ± 0.08	1.98 ± 0.28	1.344^*^	0.001

*Data were shown as mean ± standard deviation, n (%)*.

* *t-test*.

† *x2 value*.

*AHI, apnea hypopnea index; BMI, body mass index; LSpO2 (%), lowest arterial oxygen saturation; T90%, oxygen below 90% of the time; TS90%, the percentage of the total recorded time spend below 90% oxygen saturation; ODI4, oxygen desaturation index*.

### Clinical Characteristics of Lung Cancer Patients With OSA

The clinical characteristics of the training and validation sets were evaluated. The characteristics of the 90 patients in the development cohort and 38 patients in the validation cohort are showed in Table 2. The majority of patients are men and the TNM stages were represented, there were no statistically significant difference between development cohort and validation cohort.

**Table 2:** Baseline clinical features of lung cancer patients with OSA [Mean ± SD/No (%)].

**Characteristics**	**Total** **(*n* = 128)**	**Development cohort** **(*n* = 90)**	**Validation cohort** **(*n* = 38)**	**Statistics**	***P***
Age, year	60.98 ± 2.45	60.53 ± 2.34	61 ± 1.98	1.453^*^	0.224
Sex				0.048^†^	0.827
Male	86 (67.2)	61 (67.8)	25 (65.8)		
Female	42 (32.8)	29 (32.2)	13 (34.2)		
AHI	9.87 ± 1.98	9.42 ± 1.53	10.32 ± 0.97	0.634^*^	0.543
TNM stage				2.460^†^	0.483
I	34 (26.6)	23 (25.6)	11 (28.9)		
II	45 (35.2)	31 (34.4)	14 (36.8)		
III	38 (29.7)	26 (28.9)	12 (31.6)		
IV	11 (8.5)	10 (11.1)	1 (2.7)		
Cancer types				0.547^†^	0.460
Small cell carcinoma	19 (14.8)	78 (86.7)	31 (82.6)		
Non-small cell carcinoma	109 (85.2)	12 (13.3)	7 (18.4)		
BMI	21.90 ± 2.98	22.74 ± 3.42	21.45 ± 2.54	1.425^*^	0.628
Hypertension	44 (34.4)	31 (34.5)	13 (34.2)	0.001^†^	0.979
Diabetes	42 (32.8)	30 (33.3)	12 (31.6)	0.037^†^	0.847
Coronary heart disease	27 (21.2)	20 (22.2)	7 (18.4)	0.232^†^	0.630
Heart rate, beats/min	92.34 ± 17.92	93.44 ± 16.43	91.43 ± 13.54	0.425^*^	0.087
KPS	76.34 ± 8.342	74.55 ± 9.43	79.54 ± 6.74	1.445^*^	0.154
Smoking history	57 (44.5)	42 (46.7)	15 (39.5)	0.559^†^	0.454
LSpO2 (%)	69.43 ± 6.31	70.87 ± 7.43	68.43 ± 5.73	1.434^*^	0.563
T90%,min	91.23 ± 4.43	87.54 ± 3.43	93.23 ± 5.43	2.543^*^	0.623
TS90%,%	22.41 ± 2.89	21.75 ± 2.54	23.21 ± 3.54	1.240^*^	0.154
ODI4	1.98 ± 0.28	1.72 ± 0.23	2.01 ± 0.35	0.643^*^	0.634

*Data were shown as mean ± standard deviation, n (%)*.

* *t-test*.

† *x2 value*.

### Biomarker Selection

All the available information including general data, clinical characteristics and biomarkers were included for univariate and multivariate analysis (Table 3). In univariate analyses age, AHI, TNM stage, types, BMI, LSpO2 (%) and ODI4 were related to OS. All of the potentially important biomarkers identified in univariate analysis were further included in the multivariate analysis. Based on 90 OSA with lung cancer patients with complete information, age, AHI, TNM stage, types, BMI and ODI4 were significant predictors of OS.

**Table 3:** Univariate and multivariate Cox hazards analysis between clinical features and OS (*n* = 90).

	**Univariate analysis**			**Multivariate analysis**		
**Variables**	**OR**	**95% CI**	***P***	**OR**	**95% CI**	***P***
Age (≥60 vs. <60)	4.523	1.432–7.546	0.008	2.543	1.053–5.324	0.021
Sex (male vs. female)	2.435	0.234–3.253	0.454			
AHI (≥15 vs. <15)	5.434	2.432–7.545	0.032	3.245	1.323–5.435	0.006
TNM stage (≥3 vs. <3)	8.655	4.345–9.553	0.027	2.431	1.634–4.523	0.005
Cancer types (small cell carcinoma vs. non- small cell carcinoma)	1.321	1.023–4.328	0.008	1.043	1.002–2.431	0.038
BMI (≥24 vs. <24)	2.341	1.453–4.523	0.012	1.532	1.332–3.454	0.007
Hypertension (yes vs. no)	2.412	0.453–4.545	0.673			
Diabetes (yes vs. no)	1.167	0.446–1.438	0.098			
Coronary heart disease (yes vs. no)	1.432	0.314–1.634	0.342			
Heart rate (<90 vs. ≥90 beats/min)	1.342	0.754–2.525	0.234			
KPS (<90 vs. ≥90)	1.554	0.186–2.345	0.423			
Smoking history (yes vs. no)	1.543	0.423–2.253	0.564			
LSpO2% (60 vs. <60%)	1.234	1.134–4.323	0.006	1.543	0.156–3.234	0.078
T90% (≥60 vs. <60 min)	2.421	0.543–2.232	0.743			
TS90% (≥80 vs. <80%)	1.432	0.453–1.354	0.355			
ODI4 (≥15 vs. <15)	1.543	1.023–4.323	0.046	1.554	1.043–2.456	0.031

### Development of the Prediction Model

**Figure 1:**
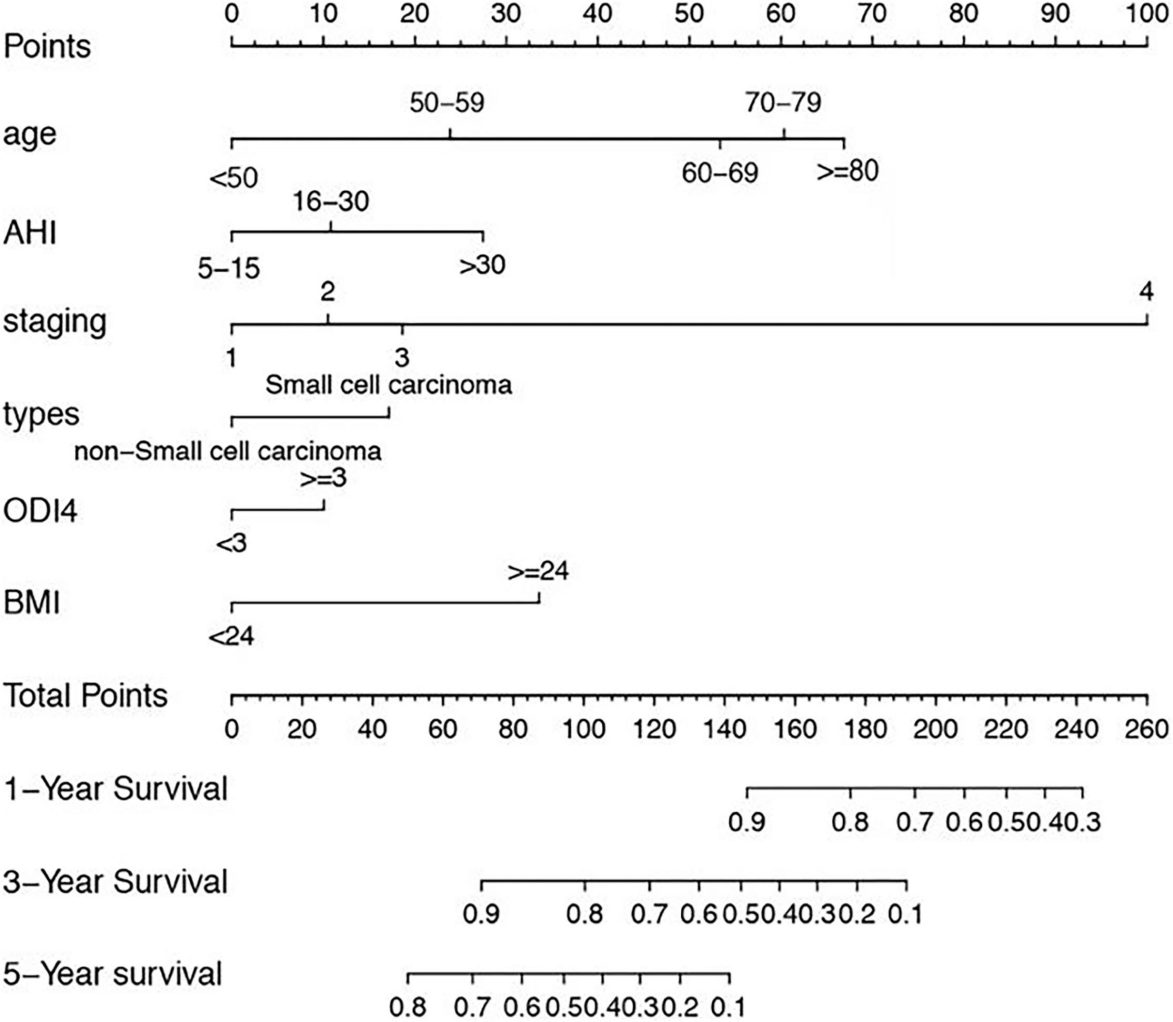
Nomogram, including Age, AHI, TNM stage, types, BMI and ODI4 for 1, 3, and 5 years overall survival (OS) in lung cancer patients with OSA. The nomogram is valued to obtain the probability of 1, 3, and 5 years survival by adding up the points identified on the points scale for each variable.

A nomogram is a graphic representation of the solution of an equation that provides a reasonable approximation of the probability of a particular outcome, nomogram was developed to predict for survival using the six independent covariates identified in the multivariate model, the mode explanatory covariables consisted of age, AHI, TNM stage, types, BMI and ODI4. A nomogram was constructed to predict 1-, 3-, and 5-year OS (Figure 1).

### Validation of the Predictive Accuracy of Nomograms for OS

**Figure 2:**
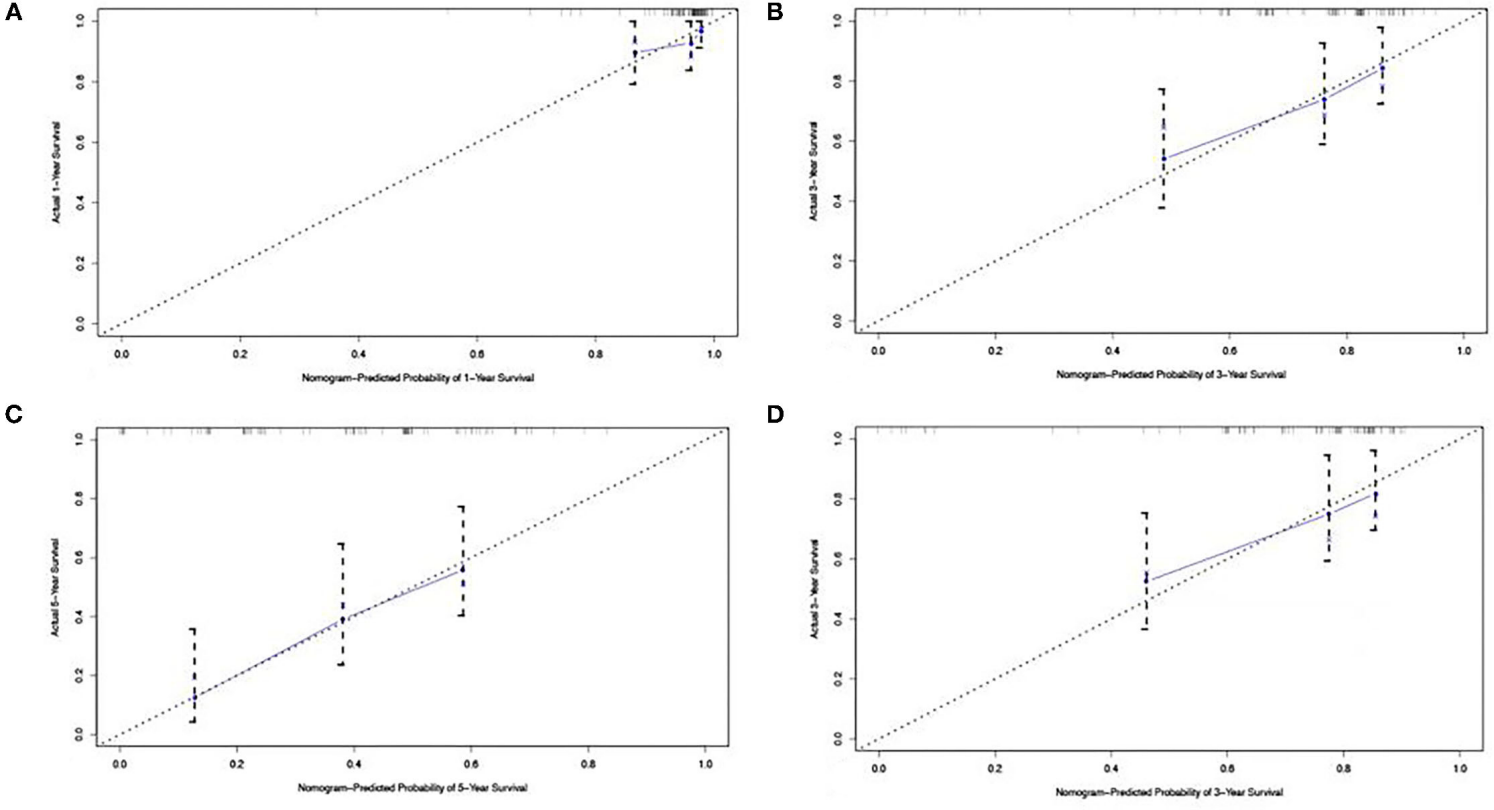
The calibration curve for predicting patient survival at **(A)** 1 year, **(B)** 3 years, and **(C)** 5 years in the development cohort and at **(D)** 3 years in the validation cohort. Nomogram-predicted probability of overall survival is plotted on the *x*-axis; actual overall survival is plotted on the *y*-axis.

After internal verification, the C-index of the nomogram model for OS prediction was 0.802 (95% CI 0.767–0.885). The calibration curve illustrates how the predictions from the nomogram compare with actual outcomes for the 90 patients. The dashed line represents the performance of an ideal nomogram, in which predicted outcomes mostly match with the actual outcomes. The dots were calculated from sub-cohorts of our dataset and represent the performance of our nomogram based on the six biomarkers of Cox model. The calibration plot for the probability of OS at 1, 3, or 5 years after therapy showed an optimal agreement between the prediction by nomogram and actual observation (Figure 2).

### ROC of the Predictive Accuracy of Nomograms for OS

**Figure 3:**
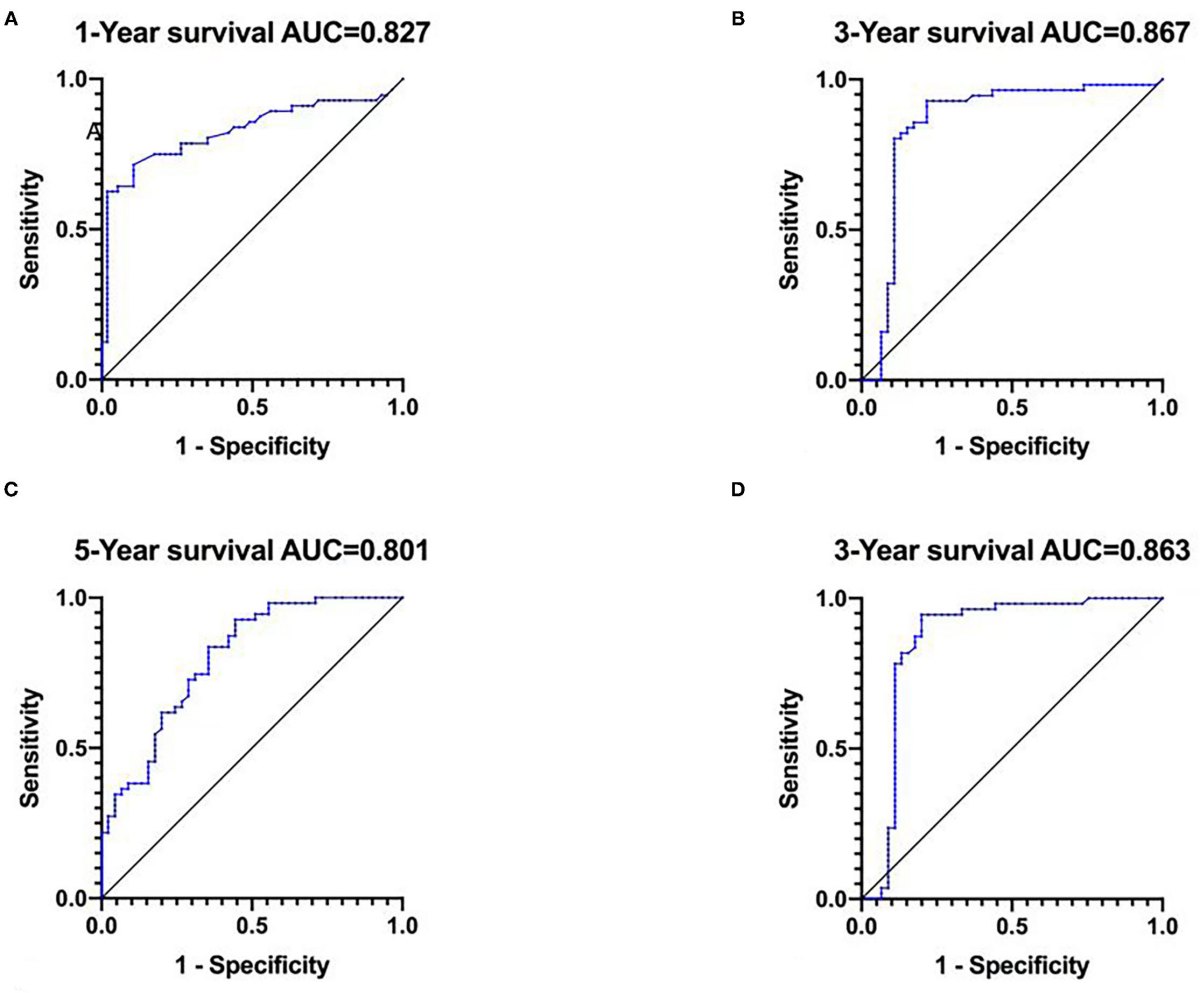
The ROC curve for predicting patient survival at **(A)** 1 year, **(B)** 3 years, and **(C)** 5 years in the development cohort and at **(D)** 3 years in the validation cohort.

The ROC curve analysis for the nomogram, area under curve (AUC) is used to evaluate the accuracy of the model, the higher the AUC value, the better the model effect. Results show 1-year survival (AUC = 0.827), 3-year survival (AUC = 0.867), 5-year survival (AUC = 0.801) in the development cohort and 3-year survival (AUC = 0.863) in the validation cohort were with good accuracy (13) (Figure 3).

### Clinical Application of Prognostic Nomogram

**Figure 4:**
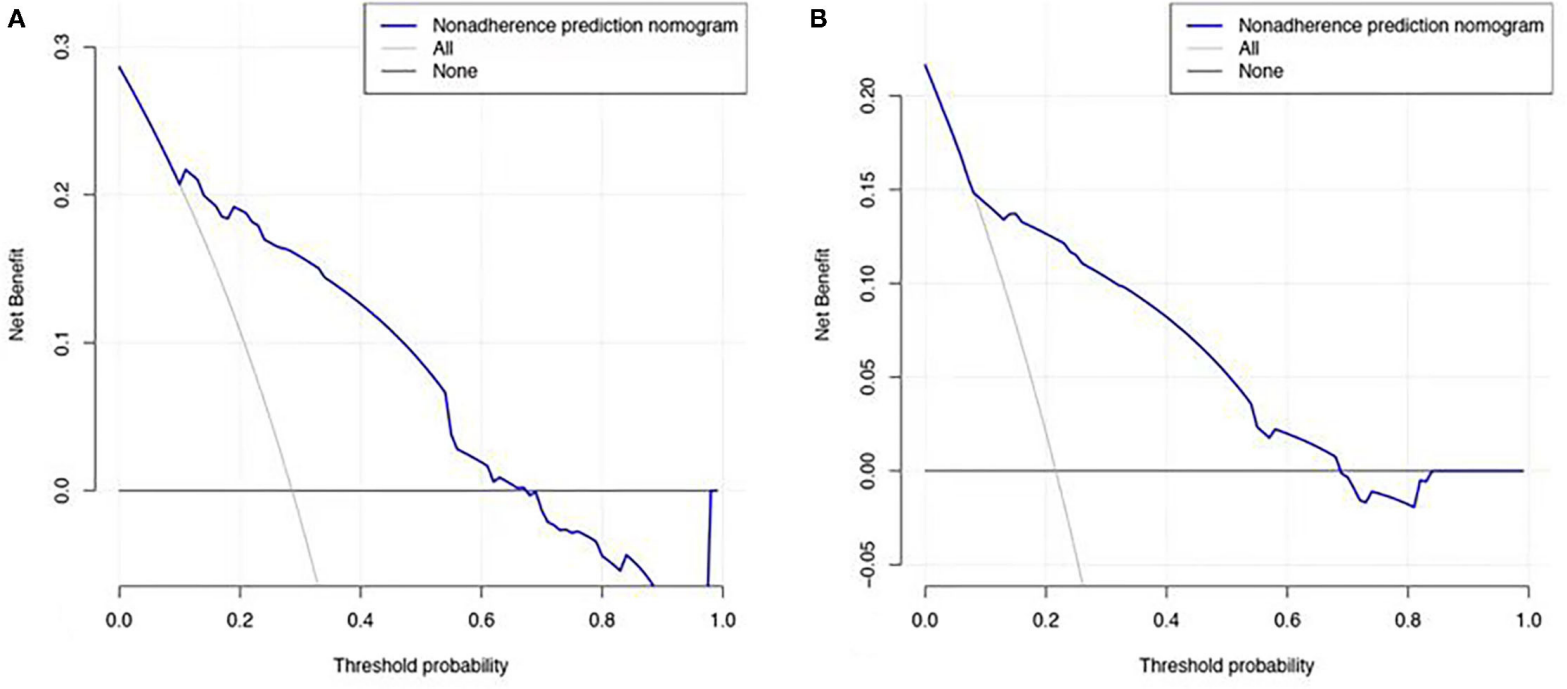
Decision curve analysis for overall survival. **(A)** In the development cohort. **(B)** In the validation cohort. The Black line: no effect of relevant independent factors. Gray line: effect of relevant independent factors. The Blue dashed line: the model of nomogram.

Decision curve analysis (DCA) is used to evaluate the predictive effect of nomogram on OS, it is very important for clinical decision-making to accurately judge the impact of clinical features and related indicators on the prognosis of lung cancer patients with OSA (14). Factors not related to prognosis were represented by black line, factors related to prognosis were represented by gray line, the model of nomogram represented by the blue line. Results show net benefit can be obtained when make decisions by nomogram (Figure 4).

**Figure 5:**
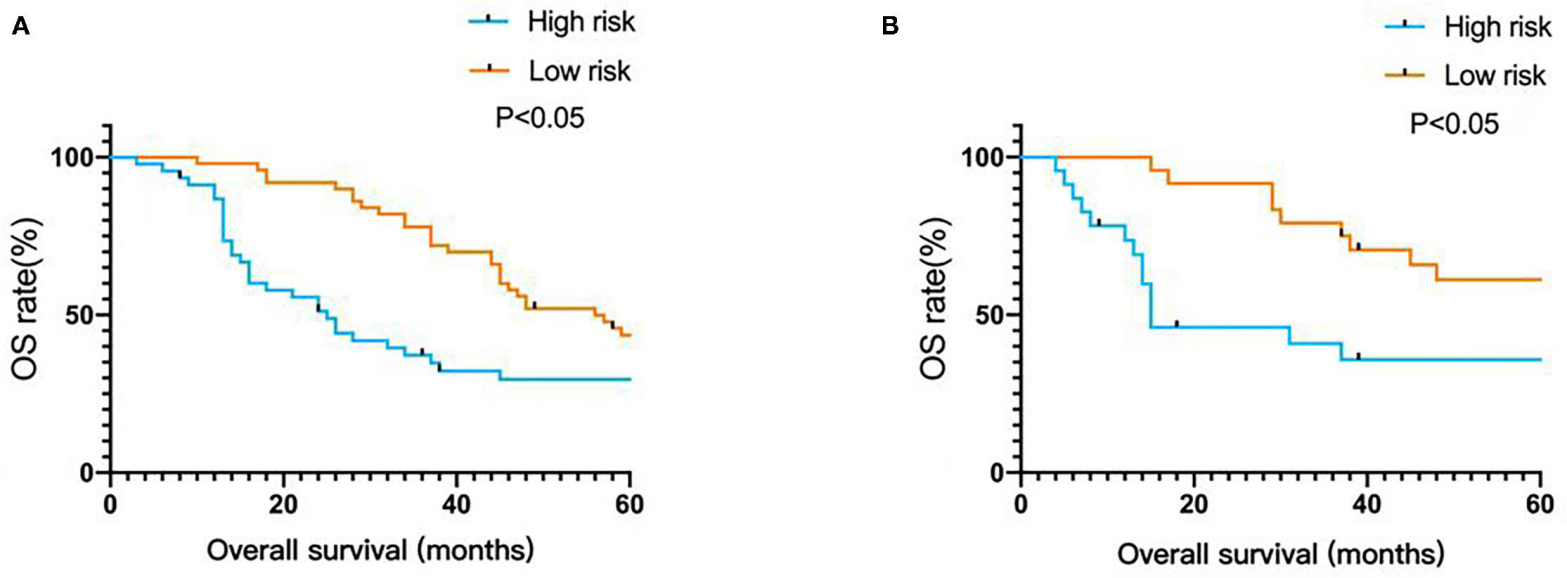
Kaplan-Meier survival curves of nomogram. **(A)** In the development cohort. **(B)** In the validation cohort.

Using this prediction model to calculate the total score, the median of the total score of all patients was 137, total score ≥137 belong to high-risk group, total score <137 belong to low-risk group. Kaplan-Meier was used to analyze OS survival in low-risk group and high-risk group, results show there was statistically significant difference in high risk group and low risk group (*P* < 0.05), the model has good accuracy and practicability (Figure 5).

## Discussion

Prognostic models can facilitate discussion between physicians and patients, the models help to identify high-risk of OSA patients individualized treatments and clinical trials can be developed and may provide insight into the biology of disease. Nomograms have been developed to predict various clinical end points for patients with all kinds of malignancies.

In recent years, multi-national cohort studies found that OSA increases the mortality of cancer, among which lung cancer was the most common malignant tumor (15, 16), Dreher et al. proposed that the incidence of OSA in new diagnosed lung cancer was 49%, the incidence of moderate/severe OSA was 17% (17), Perez-Warnisher et al. proposed that the incidence of OSA in lung cancer patients was 77.5%, in which moderate/severe OSA accounted for 41.1% (16), which consistent with the results of this study. Li et al. found that the OS of lung cancer patients with severe OSA was lower than that of patients with mild OSA, suggesting that the occurrence and severity of OSA are risk factors to promote cancer development (18). The univariate and multivariate logistic analysis indicated that age, AHI, TNM stage, cancer types, BMI and ODI4 were risk factors for overall survival. Studies confirmed that age is an important factor in the occurrence of lung cancer and as an independent risk factor for survival and prognosis of lung cancer patients (19). The continuous increase of obstructive sleep apnea with age challenges the current theory that mortality due to obstructive sleep apnea and cardiovascular co-morbidities affect obstructive sleep apnea prevalence at an advanced age (20). Body fat effect obstructive sleep apnea syndrome severity in different age groups, the neck and waist circumferences showed a statistically significant correlation with apnea-hypopnea index in both the full sample and in the ≥40 and <60 years age group, these variables did not show any significant correlation with the other two age groups (<40 and ≥60 years) (21). We found that age is an independent risk factor for lung cancer patients with OSA, and the nomogram score increases fastest in the age range of 50–60 years. The elderly patients with lung cancer complicated with OSA should be closely monitored to prevent the occurrence of disease-related complications.

Apnea hypopnea index (AHI) was the gold standard for the diagnosis of OSA. AHI refers to the average number of apnea and hypoventilation per hour during sleep. AHI also a standard for grading the severity of OSA. The results suggest that the higher AHI, the lower the survival rate of lung cancer patients with OSA and the hypoxia microenvironment promotes the growth of lung tumors. This conclusion has been confirmed by relevant studies. Stimulating the intermittent hypoxia in patients with OSAS induce pulmonary metastasis of melanoma (22), other evidence also suggests that hypoxic microenvironment contributing the development of non-small cell lung cancer (23). *In vitro* studies further proved that intermittent hypoxia lung cancer cells are more resistant and more prone to metastasis (24), indicating that lung cancer and OSAS promote each other, leading to disease progression and reduced survival.

Li et al. reported that tumor staging is related to the severity of OSA, jointly effect the prognosis of lung cancer patients with OSA (18). This study explored the effect of TNM stage to the prognosis, results show TNM stage was an independent risk factor for the prognosis of lung cancer patients with OSA. In addition, the nomogram score of patients with TNM stage IV increased significantly. TNM stage was not simple linear relationship with OS and prognosis, we should pay attention to the progress of the disease of patients with advanced lung cancer, actively treatment intervention to slow down the progress of the disease and improve the quality of life. According to histological classification, lung cancer can be divided into small cell lung cancer (SCLC) and non-small cell lung cancer (NSCLC), non-small cell lung cancer accounts for 80% of lung cancer. After multi-disciplinary comprehensive treatments in recent years, the 5-year survival rate has been greatly improved. However, only 20–30% of patients were in the early stage when diagnosed, so most of them fail to carry out standardized treatment in time and delayed the best treatment period (25). This study analyzed the prognosis of lung cancer patients with OSA, results show the prognosis of patients with small cell lung cancer was poor and the survival time was significantly reduced, although small cell lung cancer had a good response to treatment, it was often too late to make radical resection.

Obesity is one of the most important risk factors for OSA. The results of this study show BMI is an independent prognostic factor for lung cancer patients with OSA. Obesity not only aggravates the severity of OSA, but also reduces the OS of lung cancer patients with OSA. Obese patients with lung cancer and OSA are at high risk of death. Obesity is related to the increase of throat fat, tongue fat and volume (26, 27). Obesity patients always bear severe upper airway stenosis. Abdominal and thoracic fat weaken the longitudinal tracheal traction and pharyngeal wall tension, decrease chest wall compliance, decrease lung capacity, aggravate the severity of OSA. The increased of BMI is accompanied by an increase of the incidence of respiratory events and more severe nocturnal hypoxemia (28), indicating that the higher the degree of obesity, the higher the severity of OSA (29). However, some studies suggest that BMI effects the prognosis of lung cancer by influencing all aspects of physical fitness of the body (30). Others studies suggest that high BMI is closely related to the better OS of lung cancer patients (31). But smoking is also an important confounding factor of lung cancer, so the influence of BMI on lung cancer needs further explored.

Results of Wisconsin Cohort research show severe sleep disordered breathing increases nearly five times death risk of cancer (32). Lung cancer patients prone to combined with intermittent hypoxia, apnea and daytime sleepiness (6). This study show ODI4 was an independent risk factor for lung cancer patients with OSA. Hypoxia environment plays an important role in the development of lung cancer. On the one hand, adequate oxygenation plays an important role in maintaining the normal function of cells, tissues and organs. Hypoxia is prevalent in tumor tissues, even in the absence of severe respiratory diseases. Hypoxia is the result of high proliferation rate of cancer cells, when the speed of neovascularization is slower than that of tumor growth, the amount of oxygen for metabolism can't be provided. On the other hand, lung cancer patients prone to sleep disorders. In addition to the persistent hypoxia of tumor tissue, vascular compression also promotes intermittent hypoxia and any factors causing intermittent hypoxia and apnea can aggravate OSA.

## Conclusion

This research has some limitations. The number of samples included is limited, and the follow-up time is long, so incomplete clinical information can't be avoided. It is still necessary to carry out external verification with large sample and multi center.

In summary, age, AHI, TNM stage, cancer types, BMI and ODI4 are clinical factors affecting the prognosis of lung cancer patients with OSA. The nomogram established in this study can be used to predict the prognosis of lung cancer patients with OSA and provide help for patients to formulate individualized treatment strategies.

## Data Availability Statement

The original contributions presented in the study are included in the article/supplementary material, further inquiries can be directed to the corresponding author/s.

## Ethics Statement

The studies involving human participants were reviewed and approved by Renmin Hospital of Wuhan University Institutional Review Board. The patients/participants provided their written informed consent to participate in this study.

## Author Contributions

All authors contributed to data analysis and drafting or revising the article, gave final approval of the version to be published, and agreed to be accountable for all aspects of the work.

## Funding

This work was supported by the National Natural Science Foundation of China (Nos. 81970082 and 81770089).

## Conflict of Interest

The authors declare that the research was conducted in the absence of any commercial or financial relationships that could be construed as a potential conflict of interest.

## Publisher's Note

All claims expressed in this article are solely those of the authors and do not necessarily represent those of their affiliated organizations, or those of the publisher, the editors and the reviewers. Any product that may be evaluated in this article, or claim that may be made by its manufacturer, is not guaranteed or endorsed by the publisher.

## References

[1] Zhao GQ Li YR Wang XY Ding X Wang CY Xu W. [Differential evaluation of diagnostic criteria for pediatric obstructive sleep apnea hypopnea syndrome]. Lin Chung Er Bi Yan Hou Tou Jing Wai Ke Za Zhi. (2018) 32:12–17, 22. 10.13201/j.issn.1001-1781.2018.01.003 29798203

[2] Molero-Ramirez H Tamae Kakazu M Baroody F Bhattacharjee R. Polysomnography parameters assessing gas exchange best predict postoperative respiratory complications following adenotonsillectomy in children with severe OSA. J Clin Sleep Med. (2019) 15:1251–9. 10.5664/jcsm.7914 31538596 PMC6760392

[3] Spicuzza L Campisi R Crimi C Frasca E Crimi N. Prevalence and determinants of co-morbidities in patients with obstructive apnea and chronic obstructive pulmonary disease. Eur J Intern Med. (2019) 69:e15–6. 10.1016/j.ejim.2019.08.020 31494020

[4] Huang HY Lin SW Chuang LP Wang CL Sun MH Li HY. Severe OSA associated with higher risk of mortality in stage III and IV lung cancer. J Clin Sleep Med. (2020) 16:1091–8. 10.5664/jcsm.8432 32209219 PMC7954049

[5] Justeau G Gerves-Pinquie C Le Vaillant M Trzepizur W Meslier N Goupil F. Association between nocturnal hypoxemia and cancer incidence in patients investigated for OSA: data from a large multicenter french cohort. Chest. (2020) 158:2610–20. 10.1016/j.chest.2020.06.055 32629036

[6] Liu W Luo M Fang YY Wei S Zhou L Liu K. Relationship between occurrence and progression of lung cancer and nocturnal intermittent hypoxia, apnea and daytime sleepiness. Curr Med Sci. (2019) 39:568–75. 10.1007/s11596-019-2075-6 31346992

[7] International Bladder Cancer Nomogram C Bochner BH Kattan MW Vora KC. Postoperative nomogram predicting risk of recurrence after radical cystectomy for bladder cancer. J Clin Oncol. (2006) 24:3967–72. 10.1200/JCO.2005.05.3884 16864855

[8] Karakiewicz PI Briganti A Chun FK Trinh QD Perrotte P Ficarra V. Multi-institutional validation of a new renal cancer-specific survival nomogram. J Clin Oncol. (2007) 25:1316–22. 10.1200/JCO.2006.06.1218 17416852

[9] Wierda WG O'Brien S Wang X Faderl S Ferrajoli A Do KA. Prognostic nomogram and index for overall survival in previously untreated patients with chronic lymphocytic leukemia. Blood. (2007) 109:4679–85. 10.1182/blood-2005-12-051458 17299097

[10] Karakontaki FV Panselinas ES Polychronopoulos VS Tzioufas AG. Targeted therapies in interstitial lung disease secondary to systemic autoimmune rheumatic disease. Current status and future development. Autoimmun Rev. (2021) 20:102742. 10.1016/j.autrev.2020.102742 33333235

[11] Zhao B Ma W. Dexamethasone and number of days alive without life support in adults with COVID-19 and severe hypoxemia. JAMA. (2022) 327:682. 10.1001/jama.2021.24525 35166809

[12] Camp RL Dolled-Filhart M Rimm DL. X-tile: a new bio-informatics tool for biomarker assessment and outcome-based cut-point optimization. Clin Cancer Res. (2004) 10:7252–9. 10.1158/1078-0432.CCR-04-0713 15534099

[13] Walker SP. The ROC curve redefined - optimizing sensitivity (and specificity) to the lived reality of cancer. N Engl J Med. (2019) 380:1594–5. 10.1056/NEJMp1814951 31018068

[14] Baker SG. Decision curves and relative utility curves. Med Decis Making. (2019) 39:489–90. 10.1177/0272989X19850762 31104590 PMC6786943

[15] Martinez-Garcia MA Martorell-Calatayud A Nagore E Valero I Selma MJ Chiner E. Association between sleep disordered breathing and aggressiveness markers of malignant cutaneous melanoma. Eur Respir J. (2014) 43:1661–8. 10.1183/09031936.00115413 24659545

[16] Perez-Warnisher MT Cabezas E Troncoso MF Gomez T Melchor R Pinillos EJ. Sleep disordered breathing and nocturnal hypoxemia are very prevalent in a lung cancer screening population and may condition lung cancer screening findings: results of the prospective Sleep Apnea In Lung Cancer Screening (SAILS) study. Sleep Med. (2019) 54:181–6. 10.1016/j.sleep.2018.10.020 30580192

[17] Dreher M Kruger S Schulze-Olden S Keszei A Storre JH Woehrle H. Sleep-disordered breathing in patients with newly diagnosed lung cancer. BMC Pulm Med. (2018) 18:72. 10.1186/s12890-018-0645-1 29769049 PMC5956970

[18] Li L Lu J Xue W Wang L Zhai Y Fan Z. Target of obstructive sleep apnea syndrome merge lung cancer: based on big data platform. Oncotarget. (2017) 8:21567–78. 10.18632/oncotarget.15372 28423489 PMC5400607

[19] Choi WI Choi J Kim MA Lee G Jeong J Lee CW. Higher age puts lung cancer patients at risk for not receiving anti-cancer treatment. Cancer Res Treat. (2019) 51:1241–8. 10.4143/crt.2018.513 30653747 PMC6639216

[20] Fietze I Laharnar N Obst A Ewert R Felix SB Garcia C. Prevalence and association analysis of obstructive sleep apnea with gender and age differences - Results of SHIP-Trend. J Sleep Res. (2019) 28:e12770. 10.1111/jsr.12770 30272383

[21] Borges Pde T Silva BB Moita Neto JM Borges NE Li LM. Cephalometric and anthropometric data of obstructive apnea in different age groups. Braz J Otorhinolaryngol. (2015) 81:79–84. 10.1016/j.bjorl.2014.06.001 25497852 PMC9452205

[22] Almendros I Wang Y Becker L Lennon FE Zheng J Coats BR. Intermittent hypoxia-induced changes in tumor-associated macrophages and tumor malignancy in a mouse model of sleep apnea. Am J Respir Crit Care Med. (2014) 189:593–601. 10.1164/rccm.201310-1830OC 24471484 PMC3977714

[23] Giovannetti E Wang Q Avan A Funel N Lagerweij T Lee JH. Role of CYB5A in pancreatic cancer prognosis and autophagy modulation. J Natl Cancer Inst. (2014) 106:djt346. 10.1093/jnci/djt346 24301457 PMC3906994

[24] Liu Y Song X Wang X Wei L Liu X Yuan S. Effect of chronic intermittent hypoxia on biological behavior and hypoxia-associated gene expression in lung cancer cells. J Cell Biochem. (2010) 111:554–63. 10.1002/jcb.22739 20568121

[25] Yang L Wang S Zhou Y Lai S Xiao G Gazdar A. Evaluation of the 7(th) and 8(th) editions of the AJCC/UICC TNM staging systems for lung cancer in a large North American cohort. Oncotarget. (2017) 8:66784–95. 10.18632/oncotarget.18158 28977996 PMC5620136

[26] Isono S. Obesity and obstructive sleep apnoea: mechanisms for increased collapsibility of the passive pharyngeal airway. Respirology. (2012) 17:32–42. 10.1111/j.1440-1843.2011.02093.x 22023094

[27] Pahkala R Seppa J Ikonen A Smirnov G Tuomilehto H. The impact of pharyngeal fat tissue on the pathogenesis of obstructive sleep apnea. Sleep Breath. (2014) 18:275–82. 10.1007/s11325-013-0878-4 23877124

[28] Aihara K Oga T Harada Y Chihara Y Handa T Tanizawa K. Analysis of anatomical and functional determinants of obstructive sleep apnea. Sleep Breath. (2012) 16:473–81. 10.1007/s11325-011-0528-7 21573913

[29] Jeong JI Gu S Cho J Hong SD Kim SJ Dhong HJ. Impact of gender and sleep position on relationships between anthropometric parameters and obstructive sleep apnea syndrome. Sleep Breath. (2017) 21:535–41. 10.1007/s11325-016-1413-1 27704328

[30] Oldenburg CS Boll D Nicolaije KA Vos MC Pijnenborg JM Coebergh JW. The relationship of body mass index with quality of life among endometrial cancer survivors: a study from the population-based PROFILES registry. Gynecol Oncol. (2013) 129:216–21. 10.1016/j.ygyno.2012.12.041 23296262

[31] Sepesi B Gold KA Correa AM Heymach JV Vaporciyan AA Roszik J. The influence of body mass index on overall survival following surgical resection of non-small cell lung cancer. J Thorac Oncol. (2017) 12:1280–7. 10.1016/j.jtho.2017.05.010 28552766 PMC5532070

[32] Shimoda LA Semenza GL. HIF and the lung: role of hypoxia-inducible factors in pulmonary development and disease. Am J Respir Crit Care Med. (2011) 183:152–6. 10.1164/rccm.201009-1393PP 21242594 PMC3159088

